# The priority review voucher: a misconceived quid pro quo

**DOI:** 10.1136/bmjgh-2024-015933

**Published:** 2024-12-03

**Authors:** Piero Olliaro, Els Torreele

**Affiliations:** 1 ISARIC, Pandemic Sciences Institute, Nufflield Departmente of Medicine, University of Oxford, Oxford, UK; 2 Independent Researcher, Geneva, Switzerland

**Keywords:** Global Health, Public Health

**WHAT IS ALREADY KNOWN ON THIS TOPIC**

- The US Food and Drug Administration (FDA) Priority Review Voucher (PRV) is one of a set of push-and-pull mechanisms to incentivise investments into medical innovation for otherwise neglected, non-profitable diseases for pharmaceutical companies and investors, but its effectiveness has been questioned.

**WHAT THIS STUDY ADDS**

- This article shows that the PRV, except few examples, has largely failed to deliver medical benefits for patients suffering from neglected diseases because it rewards obtaining FDA marketing authorisation without regard for the products actually being available, affordable and equitably accessible for people.

**HOW THIS STUDY MIGHT AFFECT RESEARCH, PRACTICE OR POLICY**

- This article highlights shortcomings in the design and use of PRVs that make them unsuitable to broader adoption by other countries.

Various ‘push-and-pull’ mechanisms have been tried to attract pharmaceutical companies into medical innovation for public health priorities, which they, otherwise, disregard as being not profitable enough. They include public funding of basic and clinical research and a set of incentives, such as tax credits, reimbursement schemes, advanced market commitments, stockpiling, orphan drug status, intellectual property protection and regulations.^1^

The United States Food and Drug Administration (US FDA) Priority Review Voucher (PRV) programme is a regulatory incentive originally proposed for tropical diseases,^2^ later extended to rare paediatric indications and medical countermeasures. Under the PRV programme, the applicant for US marketing authorisation of a product for one of the eligible indications receives priority review for that product. Importantly, if the product is successfully registered, the applicant is also granted a transferable PRV for another product of choice that does not itself qualify for priority review, which can be redeemed or sold to another company.^3^ The potential of a 6-month earlier market entry of a high-revenue pharmaceutical serves as a (tradable) financial incentive to invest in developing products for indications that might, otherwise, be neglected. The market value of a PRV is currently estimated at around US$100 million.^4^

It has been proposed that the European Medicines Agency (EMA) too should adopt a PRV scheme for ‘neglected disease product development’, modelled on the US FDA PRV programme.^4^ We believe that the FDA PRV programme suffers from a number of critical flaws and should not be adopted by the EMA or elsewhere for a number of reasons.

First, of 43 products awarded a PRV since 2007, few address unmet neglected disease needs and even fewer are available and accessible when and where needed, despite PRV awards being redeemed.^5^ Among the 14 products awarded the tropical disease PRV, for 4, there was no innovation as they had long been registered outside the US (artemether–lumefantrine (Coartem) for malaria; nifurtimox (Lampit) for Chagas disease; miltefosine (Impavido) for leishmaniasis and triclabendazole (Egaten) for fascioliasis), while the oral cholera vaccine (Vaxchora) is of no use in cholera-endemic countries. Paediatric benznidazole for Chagas disease was a welcome improvement and a part of the PRV financial returns is being used to increase access.^6^ Few PRV-awarded products brought a real medical benefit, such as moxidectin for onchocerciasis,^7^ fexinidazole (Fexinidazole Winthrop) for human African trypanosomiasis^8^ and two drugs for tuberculosis, bedaquiline (Sirturo) and pretomanid (Dovprela). Yet even in those cases, equitable access was not guaranteed. The high prices charged for both tuberculosis drugs have been the target of years of advocacy to make them more affordable and available in high TB burden countries.^9 10^ Furthermore, it has been argued that the PRV awarded for bedaquiline more than compensated for the (estimated) investments made by the company in its development.^11^ For moxidectin, the perspective of a PRV indeed leveraged funding by an impact investor,^7^ while paediatric benznidazole and fexinidazole, developed by the non-profit DNDi, would have likely been pursued without the PRV incentive. Neither REGN-EB3 (Inmazeb) nor mAb114 (Ebanga)—the two products awarded a PRV for medical countermeasures for filovirus diseases—which also feature in the neglected tropical diseases indication—is registered and available and affordable in Ebola virus disease-endemic countries.^12^

Second, the PRV is one mechanism within an ecosystem of push-and-pull incentives in the US for national health security priority products, including centralised financing of research and strategic stockpiling through agencies, such as the National Institute for Health and the Biomedical Advanced Research and Development Authority.^13^ These combined mechanisms have contributed to bringing to FDA registration and US national security stockpiles treatments and vaccines for high-consequences diseases—such as COVID-19, Ebola virus disease and smallpox—but they have collectively failed to ensure their wider availability and equitable access globally. The added value of the PRV is a matter of debate: an analysis of the 2020 US Government Accounting Office^3^ report concluded that the report provides ‘weak evidence of overall impact’ and shows no added value of the PRV over the other existing push-and-pull incentives.^14^ Similarly, a recent analysis of the incentive effect of tropical diseases PRVs found that ‘evidence for PRVs inducing R&D spending for tropical diseases is inconclusive, but is suggestive of either no effect or, at best, a weak (and perhaps negligible) positive effect’, while also highlighting the risk of sponsors ‘gaming the system’.^15^

Third, the main beneficiaries of PRVs are pharmaceutical companies and shareholders, while the costs of prolonged monopoly pricing and resulting profit for the voucher-using drug are borne by US taxpayers and patients globally. A purported argument in favour of the PRV is that it would ‘speed to the market’ the product that it will be used for. However, the FDA already has several mechanisms to accelerate review and patient access to drugs that are expected to bring major health benefits.^16^ Accelerating market access for products that are not considered deserving such special treatment on public health grounds goes contrary to health equity.

Adopting a PRV-like mechanism in Europe then amounts to increasing the monetary value of the incentive by extending its market entry and pricing premiums to the European market, with European citizen paying into it too.

The main argument to support the merits of the PRV as an effective incentive is the expected profits of the high-revenue product that the voucher is used for and whose marketing authorisation is accelerated.^4^ The higher the price at which that product is being sold, the better its expected profits, and the more valued the PRV as a monetary reward. This of course is an argument for the prospects of lucrative business to the pharmaceutical industry, but it is tantamount to condoning, if not encouraging, high medicine prices and is at complete odds with the public health imperative of making medicines available and affordable to all. It also ignores the ongoing societal and policy discussions in Europe around excessive pricing in the pharmaceutical sector and ways to contain those to ensure the sustainability of European healthcare.^17^

The PRV in its current format has proved to be a misconceived quid pro quo, whereby much is given for little public health return, promoting and rewarding monopoly pricing of high-revenue products, with no strings attached—which indeed makes it attractive to pharmaceutical companies and investors. However, unless addressing unmet health needs, providing therapeutic benefit and ensuring availability (including local registration), affordable pricing and access of the product awarded to the PRV becomes an integral part of the incentive programme, there is no benefit for the society, only costs. But that would require designing a very different incentive scheme.

There seems, therefore, to be no reason for a PRV-type programme for neglected diseases product development to be adopted by the EMA. It has already been pointed out that incentives provided through exceptions in regulatory mechanisms are not appropriate for antibiotics.^18^ Instead of tinkering in the margins of a market-based system that pursues financial rather than public health benefit, we need to reform pharmaceutical products R&D and pricing to serve the interests of societies and not those of investors and shareholders,^19^ as also being proposed in the context of epidemic preparedness and response.^20^

## Data Availability

All data relevant to the study are included in the article.
